# Digitizing Dignity: Analyzing Digital Twins Through the Lens of Multidimensional Human Dignity

**DOI:** 10.1111/bioe.70045

**Published:** 2025-11-01

**Authors:** Andrew J. Barnhart

**Affiliations:** ^1^ Department of Social Ethics University of Bonn Bonn North Rhine‐Westphalia Germany

**Keywords:** artificial intelligence, bioethics, digital twins, human dignity, personalized medicine

## Abstract

In precision medicine, digital twins—virtual models of patients created using personalized data and advanced machine learning—are potentially changing healthcare by predicting health outcomes and guiding medical decisions. However, their use raises complex ethical questions, particularly concerning their relationship to human dignity. Patients often regard dignity as central to their healthcare experience, and failing to incorporate this principle into the design and application of digital twins risks undermining personal autonomy, misusing sensitive data, eroding patient–provider trust, and creating broader ethical challenges. This paper argues that digital twins are not mere data sets or predictive tools, but symbolic extensions of the dignity of the individuals they represent. Using David Kirchhoffer's multidimensional framework of human dignity, this study examines how digital twins engage with both absolute (inherent) and contingent (socially constructed) dimensions of dignity. The analysis begins by exploring the multidimensional concept of human dignity, followed by a discussion of how digital twins embody these dimensions, illustrated through examples such as digital brain twins, posthumous digital representations, and disability contexts. Finally, the paper addresses the ethical implications of these findings, emphasizing the moral responsibilities of researchers, developers, and clinicians to treat digital twins as representations of patient dignity, thereby ensuring these technologies advance healthcare without compromising the fundamental respect owed to every individual.

## 1 Introduction

Artificial intelligence in medical care is beginning to manifest itself in the form of predictive digital simulation models. One of the more novel versions is digital twins. Digital twins can, at times, be difficult to define in part because of the many varieties and forms that exist. Some of the first digital twins arose for industrial purposes or for environmental studies [1, 2]. However, digital twins in medicine are, generally, virtual representations of individual patients or populations that simulate their health conditions and responses to treatments [3, 4]. They simulate and predict these health outcomes via personalized medical data and advanced analytics [3, 5, 6, 7]. The models harness a vast array of data, including genetic information, medical histories, real‐time physiological measurements, and more. The concept of digital twins extends beyond mere data representation; it involves creating dynamic models that can evolve as new data are collected, thereby providing a more comprehensive view of a patient's health condition.

In the coming years, as further advancements in artificial intelligence and machine learning take place, digital twins may further enter the medical lives of patients via precision medicine. However, despite their potential medical benefits, certain ethical implications of digital twins require exploration [8]. There are challenges related to representation and epistemic injustice, such as perpetuating or exacerbating biases and disparities in healthcare. Digital twin models often rely on datasets that may systematically marginalize certain populations, leading to a biased representation that could disadvantage these groups [7, 9, 10]. Additionally, Brent Mittelstadt highlights concerns of medical paternalism, where digital twins, if considered reliable surrogates for patient health, could undermine patient autonomy by providing seemingly objective health predictions that might shift the doctor‒patient relationship [11]. Moreover, others have emphasized the importance of consent, with some suggesting the implementation of dynamic consent models. Matthias Braun argues that dynamic consent is crucial to ensure that individuals have the ability to control how their digital twins are used, allowing them to modify or withdraw consent on the basis of the context in which their digital twins are employed [10]. However, despite all these ethical considerations, which are worthy of exploration, there has thus far been little to no consideration of digital twins in the context of human dignity, which demands careful consideration when novel biomedical technologies such as digital twins are integrated into healthcare.

Human dignity has historically been a point of reference for bioethical debates, especially debates regarding genetics, reproduction, enhancement, and end‐of‐life care [12, 13]. Some critics have argued that dignity is a useless and vague concept, meaning little more than respect for autonomy [14, 15]. But human dignity is an important and thick concept of consideration in bioethics, with multidimensional aspects that expand well beyond individual patient autonomy and respect. It touches upon aspects of human nature, society, and the various relations one has with oneself, personal standards, and avoiding humiliation [16].

Several authors have sought to clarify the many ways dignity is invoked in bioethical debate. Doris Schroeder, for instance, distinguishes four concepts of dignity: (1) Kantian dignity, the inalienable work all rational agents possess; (2) aristocratic dignity, honor tied to social rank; (3) comportment dignity, the right to public respect by conforming to social norms; and (4) meritorious dignity, dignity won by virtuous action under adversity [17]. This disambiguation explains why advocates on opposing sides of issues such as euthanasia or end‐of‐life care both appeal to dignity, yet mean quite different things. Suzy Killmister builds on Schroeder's fourfold scheme by arguing that, for practical purposes in medical ethics, these four notions collapse into just two central senses. The first is the “universal Kantian sense” of dignity that is grounded in our shared capacity for self‐legislation and inviolable human worth [16, p. 161]. The second is the “aspirational sense,” which unites both comportment and meritorious dignity as the capacity to live up to one's own standards [16, p. 161].

The current ethical literature has not sufficiently addressed the intersection of digital twins and human dignity, leaving a gap in our understanding of how digital twins interact with human dignity. Given the importance of dignity in biomedical ethical discourse, bridging this gap will be necessary as more digital twins are implemented. This paper argues that what digital twin handlers (i.e. those who design, develop, maintain, and utilize digital twins) are handling is more than raw data; what they are handling is symbols of human dignity, which then come with certain moral obligations. This will be examined through David Kirchhoffer's lens of multidimensional human dignity [12, 18, 19, 20]. This framework is very similar to Killmister's taxonomy of dignity. It considers human dignity both as absolute dignity, which emphasizes the inherent value of individuals, and as contingent dignity, which reflects the societal recognition and achievements of individuals. Determining exactly what multidimensional dignity means and why it is a meaningful and useful way of conceptualizing dignity for a digital twin context is the first task of this paper. This is followed by an exploration of how this concept translates into the digital twin context, arguing that the main ethical implication of viewing digital twins through this lens of multidimensional dignity is that researchers ought to recognize that they are handling more than raw data; what they are handling is symbols of human dignity. By applying this nuanced understanding of dignity to the context of digital twins, we can better appreciate the ethical complexities involved and ensure that the technological advancements uphold the dignity of the patients they serve.

### 1.1 The What and Why of Multidimensional Dignity

Multidimensional dignity, as explored by Kirchhoffer, presents a holistic understanding of human dignity that incorporates both absolute and contingent dimensions [18, 20, 21]. Absolute dignity refers to an inherent, indivisible, inviolable value that all human beings possess simply by virtue of being human or by possessing certain human capacities. This conception is rooted in philosophical traditions, notably those influenced by Immanuel Kant, who posited that humans have absolute worth and should always be treated as ends in themselves, not merely as a means to an end [19, 22]. Absolute dignity emphasizes that certain actions are universally unacceptable if they compromise this fundamental human worth. It asserts that every person, regardless of their condition, level of capabilities, or social status, holds intrinsic value that demands recognition and respect.

Concepts of dignity, such as species‐membership, fall into this absolute dimension of dignity. The species‐membership position posits that human dignity is inherent to all individuals simply because they belong to the human species [18, p. 377]. This suggests that there is an ontological uniqueness to being human, which bestows a fundamental worth that must be respected. This view sees dignity as an intrinsic part of our human nature, independent of individual characteristics or achievements.

The capacities perspective, another category of absolute dignity, holds that human dignity arises from specific capacities that are unique to humans, such as rationality, autonomy, conscience, or the ability to love [18, p. 377]. Crucially, it is the mere possession of these capacities, not their degree of development, that grounds dignity. Thus, infants, persons with severe disabilities, and those in altered states of consciousness retain full moral status. This view emphasizes that dignity is not a reward for achievement, but a baseline entitlement rooted in human nature. In previous work as well, Kirchhoffer refers to these as “cognitive‐affective” capacities, seeing that cognitive functions and feelings or affections are hermeneutically intertwined with each other and the world [23]. Cognition and emotion always mediate a person's perception of the world and their unique place and identity in it. But this component dimension of dignity, again, does not necessarily concern itself with the degree or the specific content of the cognitive and emotive capacities. The degree of the capacities and the content of thought, emotion, and intentionality that stems from them may change over time. But the human capacity for these is an *absolute* dimension of dignity.

On the other hand, contingent dignity is a dimension of dignity that varies according to certain conditions, achievements, or social statuses [12, 23]. Unlike absolute dignity, contingent dignity can fluctuate based on an individual's actions, moral virtues, or the recognition and respect afforded to them by society. This category of dignity is seen as more relative or earned, reflecting how individuals perceive themselves and are perceived by others in light of their accomplishments, behavior, or roles within their community [18]. Contingent dignity acknowledges the social dimensions of human worth, recognizing that while all humans possess inherent dignity, the expression and acknowledgment of dignity can be influenced by external factors and social norms. This dimension of dignity can also be found in other philosophical foundations, such as Martha Nussbaum's capabilities approach (who also borrows notions of dignity from an “Aristotelian/Marxian” view) [22, 24].

Concepts such as self‐worth and behavior views of dignity fall into the contingent dimension [12, 18]. The self‐worth view understands dignity as a sense of self‐worth or personal pride, which is developed through living a meaningful and morally good life. It emphasizes the subjective experience of dignity, where the focus is on how biomedical technologies affect an individual's self‐perception and sense of a life well lived [12, 18]. Dignity here is about achieving a meaningful existence that is recognized and respected by oneself. The behavior concept links dignity to moral behavior and a virtuous living in relation to society [12, 18]. This suggests that dignity can be acquired or demonstrated through one's actions and conduct. Dignified behavior commands admiration and respect, reflecting qualities such as composure, restraint, and moral integrity. Biomedical technologies and practices would be judged on whether they help individuals live more morally good and dignified lives.

It is important to note that these different narrow conceptions of dignity are part of a greater notion of human dignity. Hence, the consideration for human dignity should go beyond simple admiration for personal autonomy or respect for individuals—though these aspects certainly form part of the equation. A human being is not merely a species member, solely autonomous, purely a self‐reflective entity, or just a sequence of moral actions within a society. Instead, a human individual is a dynamic amalgamation of all these aspects, intertwined in an ongoing relationship over time. As Kirchhoffer describes, “A human individual is an embodied (Species membership), meaning‐seeking and meaning‐making (Self‐worth) subject (Capacities), in relation to all that is (Behavior) [18, p. 381].”

A summary table of Kirchhoffer's observed categories of dignity is found below (Table 1).

**Table 1:** Kirchhoffer's absolute and contingent categories of dignity, recreated and paraphrased.

1. Absolute dignity	2. Contingent dignity
1a. Species‐Membership: All human beings have dignity by virtue of belonging to the human species.	2a. Self‐Worth:A sense of pride in oneself, influenced by interactions with others and the world, growing through positive moral actions and diminishing through negative experiences.
1b. Capacities: All human beings have dignity by virtue of unique human capacities, despite their level of development (e.g., rationality, conscience, autonomy, love).	2b. Behavior: A realization of dignity through moral behavior, requiring recognition from others, emphasizing the social aspect of dignity.

A key reason why I adopt this version of human dignity as a lens over that of, for instance, Killmister's view is that Kirchhoffer and Dierickx applied multidimensional dignity to human tissue donation. They argue that human dignity is implied by human tissue due to its symbolic nature, although they do not state the exact meaning of the term “symbolic” [19]. For the purposes of immediate working simplicity, and perhaps to the consternation of many other scholars who work on this concept, I will take symbolic and representation to be synonymous. However, Braun does also provide a definition of representation in a digital twin context that will prove useful for this study.

Representation as it is understood here means that the simulation functions as a sort of surrogate for the simulated object or entity in a specific context: in our case, medicine. This is an especially important challenge as the simulation is, even under the condition of real‐time simulation, not located in exactly the same time and space as the represented physical entity. Thus, the term ‘representation’ is focused on the specific interaction between the embodied person and her simulated twin.

[25, p. 396]

Continuing with Kirchhoffer and Dierickx, they contend that while human tissue itself does not possess human dignity, the handling and use of such tissue in biomedical research and healthcare practices engage with the dignity of the individuals from whom the tissue originates [19]. Human tissue involves not only the genetic identity of a person but also the conscious moral decisions behind its donation. Such donations from individuals are often driven by a desire to contribute to the greater good or advance medical science. This act of donation can enhance the donor's sense of self‐worth and moral integrity, reflecting their contingent dignity. Moreover, since tissue contains genetic information that can significantly impact identity and social perceptions, its use in research must be handled with the utmost ethical consideration to avoid discrimination and misuse [19]. Thus, ethically sound treatment of human tissue exemplifies respect for both the absolute and contingent dignity dimensions of the individual. This application of multidimensional dignity to human tissue illustrates the potentially broader applicability of the concept to various biomedical technologies, such as digital twins.

Moreover, Kirchhoffer discusses the usefulness of multidimensional dignity as it relates to the issue of “dignity talk” [18]. Opposing sides in ethical debates often appeal to specific concepts of dignity that are more sympathetic or malleable to their arguments, which can lead to a moral impasse. Multidimensional dignity attempts to circumvent such impasses by acknowledging dignity as both inherent and socially constructed. This approach also allows dignity to serve both descriptive and normative functions. Descriptively, it clarifies which aspects of being human are deemed valuable by different positions in a debate. Normatively, it approves only those practices and technologies that contribute to the flourishing of persons as multidimensional wholes.

## 2 Digital Twins and Human Dignity

We must now turn our attention to the task of examining digital twins through the lens of multidimensional human dignity. In doing so, I argue that digital twin researchers and handlers are handling the symbols and representations of a patient's dignity and not mere data. To be clear, while digital twins may be virtual representations and predictive simulations of persons, they do not in and of themselves possess dignity. Digital twins, including the brain, full‐body, or psychological digital twins, are not moral agents. They do not possess cognitive capacities or a specific ontological nature that demands ascension into a moral community, let alone personhood. They do not have a sense of self, nor is there evidence that society generally views them as entities conducting dignified behavior. However, if there is dignity “within” these digital twins, it is such that it originates from the respective physical patient.

### 2.1 Absolute Dignity

The data collected to develop digital twins in healthcare are comprehensive and multifaceted, encompassing a wide range of personal health information. This includes but is not limited to genetic data, medical history, real‐time physiological measurements, and environmental exposure information [7, p. 31]. The data that the digital twin captures and analyzes can thus easily identify the patient as a (specific) human being. Digital twins exist for other entities, such as animals, machines, and cities. However, there appear to be acceptable differences between how digital twin handlers may handle a digital twin based on what the digital twin is twinning. There is an added aspect of the real‐time connection between the digital twin and the patient. While manipulating the digital twin will not directly affect the person physically, it can still have further downstream implications for that person's medical care. From the human‐species perspective, both the fact that digital twins are made from the data that necessarily identifies the patient and that digital twins are connected to the patient in real‐time are enough to conclude that digital twins ought to be handled with dignity in mind.

Digital twins in medicine focus primarily on physical health metrics and predictive analytics. Although certain human capacities cannot be fully represented (e.g., love), they have the potential to assess aspects related to the consciousness, autonomy, and rationality of patients, albeit indirectly. For example, while digital twins do not directly measure consciousness, they can monitor neurological health indicators and cognitive function through real‐time data collection, tracking changes in brain activity or responses to stimuli, which may provide insights into a patient's level of consciousness [26, 27]. However, the interpretation of consciousness itself remains a complex and highly debated area in which digital twins can only support through data rather than directly assess. Nevertheless, the capacities view of dignity emphasizes that human dignity involves both inherent capacities and the realization of these capacities in a morally meaningful life. Once again, it should be noted that the specific level of the capacities does not matter under this view. It thus does not morally matter what level of a specific capacity the digital twin is capable of measuring, representing, or predicting. Rather, it is only the fact that human beings possess the potential for these capacities that warrants human dignity. Thus, if the digital twin can detect, represent, or predict *any* level of certain human capacities, then responsibilities related to human dignity is warranted.

### 2.2 Contingent Dignity

Moreover, the data that digital twins capture (e.g., genomic data) are increasingly socially relevant for forming identity [20]. While the data can identify one as a human being (and thus worthy of protection and care), the data can also include aspects, such as racial background, gender, or health status, distinguishing individuals in ways that may impact their treatment and social perception. These perceptions can either support the individual's inherent value, as seen in the personalized medicine's aim to cater to each person's unique health requirements, or, if misused, could contribute to discrimination and harm a person's sense of self‐worth.

Furthermore, a person realizes their potential (“the dignity one has”) through moral interactions [19]. This moral pursuit of their own potential nurtures and matures a person's sense of self‐worth. The choice to share personal data for creating a digital twin could thus, at least in part, be driven by a belief in the positive impact the technology may have, either for oneself or for the broader community. This decision may mirror the ethical considerations seen in the donation of human tissue, where individuals contribute with the hope of advancing medical science and aiding others, thus reinforcing their own sense of worth and dignity.

### 2.3 Dignity Over Time

There is another dignity dimension that may be relevant for digital twins. This is what Kirchhoffer calls “being in relationship over time [18, p. 379].” All forms of dignity are relational. To be a member of the human species is to be related to all other humans to varying degrees. Capacities are related to the entities that possess them. One's self‐worth is related to oneself. In addition, dignified behavior is related to a broader community since others are needed to recognize this moral behavior. However, notably, certain dignity dimensions and concepts are dynamic in time. As Kirchhoffer states regarding capacities, “For example, one's autonomy is almost non‐existent in one's infancy, develops to a peak in adulthood, and then, for many, deteriorates as one reaches extreme old‐age” [18, p. 379]. This dynamic element of dignity is important for medical digital twins because they are also predictive models and simulations, which provide (potential) glimpses into the future self. A digital twin presents to a patient with a potential version of their future self and offers changes in their behavior (or medical treatment) that could be existentially meaningful [25]. It thus directly confronts patients about their own ideas of realizing their potential and thus their own dignity. A digital twin, therefore, becomes a symbol for both the absolute and contingent dignity of a particular patient, not only because of the kind of data that make up the digital twin but also because of the predictive outputs it generates. Moreover, the potential for confronting one's own dignity dimensions means that there will be influence on the choices the patient may make.

## 3 Ethical Implications

Given both the number of ways dignity is conceived in multidimensional human dignity and the many forms and applications digital twins have, there will inevitably be a great many ethical implications. To help bring theory and practice closer together, I present three illustrative cases and contexts: a digital brain twin example, which forces individuals to confront predictions of diminished capacities and threatened sense of self‐worth; the issue of dignity after death, which considers implications of consent, respect, governance strategies of post‐mortem digital likenesses; and disability, which highlights the risk of bias and the ethical imperative to model embodied impairments with nuance and respect. Together, these cases reveal how digital twins represent both absolute and contingent dimensions of dignity and show just some of the ethical implications that they entail.

### 3.1 Digital Brain Twin Example

For a slightly more concrete example regarding dignity over time, let us suppose that a researcher generates a digital brain twin of a patient whose medical history and genome were indicated in prior testing for an elevated risk of developing late‐stage Parkinson's disease. This digital brain twin could be generated not only from genomic data and patient medical history, but also from data gathered via a breathing belt worn at night or from wireless radio signals, which bounce off the patient's body to detect breathing patterns [28].^1^ It then processes these breathing patterns via neural networks and graphics processing to create a digital brain twin with the ability to detect the condition and predict its development over time.

Suppose then that the digital brain twin developed in this way for a patient detects the presence of Parkinson's disease and predicts a severe onset of the condition approximately 10 years from now. The digital twin presents this information not only by means of a prognosis readout but also in a virtual representation of the patient's future brain, with which the patient can visually interact. The digital twin may also provide simulation outcomes for variations in medical treatments and interventions. The digital twin prognosis may indicate that although there is no cure for Parkinson's disease, combinations of pharmacotherapy, physiotherapy, and good care management are helpful in alleviating symptoms of disease progression [29]. The digital twin may indicate that there will be a decrease in certain capacities over time.

The patient must now confront what the digital twin represents in the context of their dignity. They may interpret that the potential for diminished capacities of rationality, autonomy, and conscious awareness will lower their self‐worth and social perceptions of themselves in the future. They may thus conclude that their future dignity, represented (in part) by the digital twin, is also greatly diminished to a point where the other dimensions of dignity (e.g., the dignity of being a human being) are no longer sufficient. In light of these findings, the patient might choose an optimal point later in time (perhaps with the help of the digital twin) to commit to euthanasia or physician‐assisted suicide; to “die with dignity.” However, in this sense, to die with dignity could mean preserving a sense of self‐worth (self‐worth concept) and being remembered in a more socially positive manner (behavior concept). This is not to say that all patients will respond to the confrontation of their perceived potential future dignity in the same way, but it is not hard to imagine that such responses may arise.

The digital brain twin in this case represented not only various dignity dimensions of the patient in the present moment but also the dignity of the patient in the future, which forced the patient to confront their future dignity dimensions. The point of this case is not about whether the patient responded morally or appropriately to the confrontation about their future dimensions of dignity that the digital twin brought about. Rather, it shows that digital twins can and do represent our dignity, which will bring about such confrontations, and it is this that is worthy of deeper moral consideration from all agents involved with digital twins in medicine. It calls for careful controls on how prognostic simulations are framed and delivered, rigorous (yet perhaps dynamic) informed‐consent processes that anticipate emotional impacts, and support mechanisms (e.g., counseling) to buffer against fatalistic or stigmatizing interpretations.

### 3.2 Dignity After Death

Furthermore, even after the death of the patient, there is a question of how to appropriately handle the digital twin that respects the dignity of the departed person. As Iqbal, Krauthammer, and Biller‐Andorno noted, digital twins may change the way in which we understand the nature of end‐of‐life [30]. Medicine often ties the person to the body, and bioethics explores very little about post‐death issues, with the exceptions of organ or body donation and a person's digital afterlife in relation to social media [30, 31, 32]. Digital twins require us to think past the death of the body since the digital model will remain. This, in turn, raises important questions. Do we handle the remaining digital twins in a similar fashion to the physical remains? Much like organ donation, do we need to create similar consent structures for donating the digital twin to science? Alternatively, should the remaining digital twin be treated more like how we treat public and personal forms of data (e.g., emails, social media profiles, personal writings in computer files)?

However, even in the digital afterlife, dignity still plays a role in the governance of digital twins. As Öhman and Floridi alluded in discussions on the digital afterlife industry, “Ethically, human dignity requires that digital remains, seen as the informational corpse of the deceased, may not be used solely as a means to an end, such as profit, but regarded instead as an entity holding an inherent value” [31, p. 319]. As I stated, digital twins do not themselves have dignity that is distinct from the physical person they represent. Their inherent value comes from their ability to symbolize the various dimensions and concepts of dignity related to their physical counterparts. However, as Öhman and Floridi noted, treating a deceased person's digital twin with dignity would entail treating the digital twin as something akin to an informational corpse of the person. This would mean that handlers nevertheless must consider the absolute and contingent dimensions of dignity within this context.

Barnhart, Comerci, and Braun have also identified other normative notions involved when considering decommissioning strategies for digital doppelgängers (similar to digital twins). The way digital twins ought to be decommissioned (archival, repurposing, or destruction) will also depend upon whether they are viewed as a proxy or as an extension of personhood [33]. From a proxy viewpoint, archival can rely on lower minimal standards (much like preserving an owned artifact), repurposing is broadly permissible, and destruction carries little expectation of ceremonial closure. By contrast, treating a twin as an extension of personhood demands robust archival protocols (a virtual “cemetery” with strict access and preservation criteria), tight limits on repurposing to safeguard the deceased's identity and legacy, and ritualized destruction that honors the twin as the “informational corpse” of the represented person. Embedding these distinctions into advance‐directive consent forms and institutional policies allows for each decommissioning pathway to respectfully balance the absolute and contingent dimensions of dignity at stake.

### 3.3 Disability and Dignity

As another example, digital twins have the potential to serve the needs of individuals with disabilities and impairments. Such groups historically have been significant stakeholders within the human dignity debate in medicine, often engaging in “dignity talk.” Consider the idea of constructing a digital twin for a person with a leg amputation. How shall this digital twin represent the embodiment of the impairment for this person, and to what aim? There are, after all, multiple ways in which the amputation can be embodied, such as with a prosthetic, with only crutches, or with a wheelchair. Amputees often will move between these different modes of embodiment, shifting from crutches to a wheelchair if they feel tired, for example [34].

The absolute dimensions of dignity that pertain to all human beings must be considered. These forms of dignity, which are inherent and cannot be lost, require that the person be treated with equal respect regardless of her disability. A digital twin that is used to enhance her mobility and quality of life could align with this by affirming her equal worth and right to access technologies that improve her well‐being. However, if the digital twin is used in ways that perpetuate societal biases or inequalities—such as prioritizing certain modes of embodiment (e.g., prosthetics) over others (e.g., wheelchairs) based on normative assumptions about what constitutes “normal” mobility—it could violate her dignity by marginalizing her lived experience as an amputee.

There is also the risk that such a digital twin could undermine her dignity if it reduces her to a set of data points or focuses solely on functional optimization without considering her broader identity. For example, if the digital twin prioritizes efficiency over her personal preferences or fails to account for the emotional and relational aspects of her embodiment, it could inadvertently diminish her sense of self‐worth. This tension further highlights the fragility of dignity as a sense of self‐worth, which can be easily compromised by external factors, such as the design and use of technology.

What all this implies is that designers must have inclusive representations that respect each individual's embodiment choices, avoid stigmatizing language or default assumptions about (in this case) “optimal” mobility, and balance performance optimization with the emotional, relational, and identity‐affirming dimensions of dignity. In practice, this means bringing people with impairments and disabilities in as co‐designers, embedding preferences into simulation parameters, and auditing models for hidden biases that could erode the different dimensions of dignity.

### 3.4 Limitations

Despite the conceptual robustness of the multidimensional human dignity framework, its practical application to digital twins in medicine is hampered by certain challenges. One such challenge is contending with the moral weight of divergent dignity dimensions. Faced with such a tension, one could be tempted to adopt one of three default moves: defer entirely to the patient's own preference as the final moral arbiter; insist on primacy of absolute dignity, rejecting rationale in contingent dignity dimensions; or dismiss the very notion of assigning “moral weight” to competing dignity claims as an ill‐founded exercise in qualification. Yet each move carries its own perils: privileging personal preference risks reinforcing internalized stigma, absolutism may overlook self‐regarding aspirations, and abandoning moral weight altogether threatens to trivialize the genuine ethical tensions at stake. A possible way to handle this challenge of moral weight would be to examine moral weight as an empirical, context‐sensitive variable that should be discovered through iterative, practice‐based inquiry. By piloting different balancing strategies in real clinical cases, gathering feedback on patient well‐being and moral distress, and continuously revising guidelines in light of what actually promotes flourishing and trust, we allow the moral weight of differing human dignity dimensions to emerge as a fallible yet responsive tool.

Moreover, the ethical and moral analysis of digital twins does not, and should not, end with human dignity. While multidimensional dignity gives us insights into how digital twins symbolically confer the various aspects of human dignity, digital twins also raise distinct concerns about data accuracy and reliability, transparency and explainability, consent and control, equity of access, responsibility and liability, ontological ambiguity, behavioral influence, manipulation, and environmental impact [10, 25, 30]. These areas will require different ethical approaches than what dignity can offer, though human dignity can help inform them.

## 4 Conclusions

What we find in this exploration is that navigating dignity can be a complex endeavor. But moral and ethical complexity does not mean such situations are unnavigable, or that our ethical lenses are impractical. Descriptively, human dignity can be related to digital twins by examining how digital twins represent and interact with human identity, autonomy, and moral worth. In this descriptive function, dignity helps to identify and articulate the values and aspects of humanity that are considered most important in ethical debates. The normative function of human dignity is grounded in the idea that human beings are multidimensional wholes—complex, embodied, meaning‐seeking, and relational beings. This understanding of dignity requires that any action, including the handling of digital twins, must respect and promote the flourishing of individuals as these multidimensional beings. The normative function, therefore, does not allow for a simplistic focus on one aspect of humanity (e.g., autonomy or species membership) at the expense of others. Instead, it insists that ethical decisions must consider the whole person and their relationships to others, society, and the world. Interactions with digital twins are inherently ethical decisions shaped (in part) by societal norms of right and wrong. The ethical significance arises not only from the digital replication process itself, such as how mundane digital data management may lack certain ethical considerations, but also from the critical role that digital twins can play in patient care and medical research.

The essence of digital twins, embodying both the physical and physiological data of patients, goes beyond mere digital constructs to symbolize the very individuals they represent. Moreover, the added predictive nature of digital twins means that they can symbolize the near‐countless future selves of a patient. Engaging with digital twins thus engages with the essence of patient identity and dignity, treating them more than as mere data points. This engagement touches upon the dual nature of dignity—both the inherent worth of a person and the contingent aspects rooted in the trust and expectations placed in the medical system's ethical use of their information. Digital twin handlers must understand that what they are handling is not only data but also the dignity of the patient. A patient entrusts digital twin handlers with aspects that make up both their absolute dignity (e.g., genetic and physiological information) and their contingent dignity (e.g., motivations for giving the information).

Multidimensional dignity in biomedical contexts extends beyond Kirchhoffer and Dierickx's initial examination of human tissue. As digital twins increasingly influence medical decision‐making and patient interactions, the multidimensional understanding of dignity offers a valuable pillar for assessing their impact on individual and collective perceptions of worth and respect. It challenges us to ensure that such innovations not only advance healthcare but also do so in a manner that fundamentally respects and enhances the dignity of those they aim to serve.

## Conflicts of Interest

The author declares no conflicts of interest.

## Endnotes

1 See, for example, Yang et al. [28], who have noted that the relationship between Parkinson's disease and breathing patterns was first established in 1817 by James Parkinson, and thus, there is a long history of research regarding this relationship.

## References

[1] G. S. Blair , “Digital Twins of the Natural Environment,” Patterns 2, no. 10 (2021): 100359. 34693377 10.1016/j.patter.2021.100359 PMC8515001

[2] F. Tao and Q. Qi , “Make More Digital Twins,” Nature 573, no. 7775 (2019): 490–491. 31554984 10.1038/d41586-019-02849-1

[3] E. Katsoulakis , Q. Wang , H. Wu , et al., “Digital Twins for Health: A Scoping Review,” Npj Digital Medicine 7, no. 77 (2024): 77. 38519626 10.1038/s41746-024-01073-0 PMC10960047

[4] D. Jones , C. Snider , A. Nassehi , J. Yon , and B. Hicks , “Characterising the Digital Twin: A Systematic Literature Review,” CIRP Journal of Manufacturing Science and Technology 29 (2020): 36–52.

[5] R. Laubenbacher , B. Mehrad , I. Shmulevich , and N. Trayanova , “Digital Twins in Medicine,” Nature Computational Science 4, no. 3 (2024): 184–191. 38532133 10.1038/s43588-024-00607-6 PMC11102043

[6] F. S. De Sio and G. Mecacci , “Four Responsibility Gaps With Artificial Intelligence: Why They Matter and How to Address Them,” Philosophy & Technology 34, no. 4 (2021): 1057–1084.

[7] K. Bruynseels , F. Santoni De Sio , and J. Van Den Hoven , “Digital Twins in Health Care: Ethical Implications of an Emerging Engineering Paradigm,” Frontiers in Genetics 9, no. 31 (2018): 1–11. 29487613 10.3389/fgene.2018.00031 PMC5816748

[8] D. Helbing and J. A. Sánchez‐Vaquerizo , “Digital Twins: Potentials, Ethical Issues, and Limitations,” in Handbook on the Politics and Governance of Big Data and Artificial Intelligence, eds. A. Zwitter and O. Gstrein (Edward Elgar Publishing, 2023), 64–104.

[9] M. K. Cho and N. Martinez‐Martin , “Epistemic Rights and Responsibilities of Digital Simulacra for Biomedicine,” American Journal of Bioethics 23, no. 9 (2023): 43–54. 10.1080/15265161.2022.2146785 PMC10258225 36507873

[10] M. Braun , “Ethics of Digital Twins: Four Challenges,” Journal of Medical Ethics 48, no. 9 (2022): 579–580. 34380743 10.1136/medethics-2021-107675

[11] B. Mittelstadt , “Near‐Term Ethical Challenges of Digital Twins,” Journal of Medical Ethics 47, no. 6 (2021): 405–406. 10.1136/medethics-2021-107449 33963064

[12] D. G. Kirchhoffer , Human Dignity in Contemporary Ethics (Teneo Press, 2013).

[13] A. Schulman , “Bioethics and the Question of Human Dignity,” in Human Dignity and Bioethics, ed. B. T. Lanigan (Nova Science Publishers, Inc., 2009), 3–14.

[14] A. Cochrane , “Undignified Bioethics,” Bioethics 24, no. 5 (2010): 234–241. 20002071 10.1111/j.1467-8519.2009.01781.x

[15] R. Macklin , “Dignity Is a Useless Concept,” BMJ 327, no. 7429 (2003): 1419–1420. 14684633 10.1136/bmj.327.7429.1419 PMC300789

[16] S. Killmister , “Dignity: Not Such a Useless Concept,” Journal of Medical Ethics 36, no. 3 (2010): 160–164. 20211996 10.1136/jme.2009.031393

[17] D. Schroeder , “Dignity: Two Riddles and Four Concepts,” Cambridge Quarterly of Healthcare Ethics 17, no. 2 (2008): 230–238. 18312738 10.1017/S0963180108080262

[18] D. G. Kirchhoffer , “Human Dignity and Human Enhancement: A Multidimensional Approach,” Bioethics 31, no. 5 (2017): 375–383. 28182276 10.1111/bioe.12343

[19] D. G. Kirchhoffer and K. Dierickx , “Human Dignity and Human Tissue: A Meaningful Ethical Relationship?,” Journal of Medical Ethics 37, no. 9 (2011): 552–556. 21478417 10.1136/jme.2010.041509

[20] D. G. Kirchhoffer , “Become What You Are: On the Value of the Concept of Human Dignity as an Ethical Criterion in Light of Contemporary Critiques,” Bijdragen: Tijdschrift Voor Filosofie en Theologie 70, no. 1 (2009): 45–66.

[21] D. G. Kirchhoffer , “Introduction,” in Human Dignity in Contemporary Ethics (Teneo Press, 2013), 1–45.

[22] P. Formosa and C. Mackenzie , “Nussbaum, Kant, and the Capabilities Approach to Dignity,” Ethical Theory and Moral Practice 17, no. 5 (2014): 875–892.

[23] D. G. Kirchhoffer , “Chapter 3: The Component Dimensions of Human Dignity Model,” in Human Dignity in Contemporary Ethics (Teneo Press, 2013), 205–253.

[24] M. C. Nussbaum , “Human Dignity and Political Entitlements,” in Human Dignity and Bioethics, eds. B. T. Lanigan (Nova Science Publishers, Inc., 2009), 245–264.

[25] M. Braun , “Represent Me: Please! Towards an Ethics of Digital Twins in Medicine,” Journal of Medical Ethics 47, no. 6 (2021): 394–400. 10.1136/medethics-2020-106134 33722986

[26] Y. Takahashi , H. Idei , M. Komatsu , J. Tani , H. Tomita , and Y. Yamashita , “Digital Twin Brain Simulator for Real‐Time Consciousness Monitoring and Virtual Intervention Using Primate Electrocorticogram Data,” Npj Digital Medicine 8, no. 80 (2025): 1–13. 39929926 10.1038/s41746-025-01444-1 PMC11811282

[27] H. E. Wang , P. Triebkorn , M. Breyton , et al., “Virtual Brain Twins: From Basic Neuroscience to Clinical Use,” National Science Review 11, no. 5 (2024): nwae079. 38698901 10.1093/nsr/nwae079 PMC11065363

[28] Y. Yang , Y. Yuan , G. Zhang , et al., “Artificial Intelligence‐Enabled Detection and Assessment of Parkinson's Disease Using Nocturnal Breathing Signals,” Nature Medicine 28, no. 10 (2022): 2207–2215. 10.1038/s41591-022-01932-x PMC9556299 35995955

[29] B. R. Bloem , M. S. Okun , and C. Klein , “Parkinson's Disease,” Lancet 397, no. 10291 (2021): 2284–2303. 33848468 10.1016/S0140-6736(21)00218-X

[30] J. D. Iqbal , M. Krauthammer , and N. Biller‐Andorno , “The Use and Ethics of Digital Twins in Medicine,” Journal of Law, Medicine & Ethics 50, no. 3 (2022): 583–596. 10.1017/jme.2022.97 36398633

[31] C. Öhman and L. Floridi , “An Ethical Framework for the Digital Afterlife Industry,” Nature Human Behaviour 2, no. 5 (2018): 318–320. 10.1038/s41562-018-0335-2 30962597

[32] R. B. Freeman and J. L. Bernat , “Ethical Issues in Organ Transplantation,” Progress in Cardiovascular Diseases 55, no. 3 (2012): 282–289. 23217432 10.1016/j.pcad.2012.08.005

[33] A. J. Barnhart , G. Comerci , and M. Braun , “Bytes the Dust: Normative Notions in Decommissioning Digital Doppelgängers,” American Journal of Bioethics 25, no. 2 (2025): 126–129. 10.1080/15265161.2024.2441708 PMC11789699 39878738

[34] M. K. Nelson , A. Shew , and B. Stevens , “Transmobility: Possibilities in Cyborg (Cripborg) Bodies,” Catalyst: Feminism Theory, Technoscience 5, no. 1 (2019): 1–20.

